# Augmented Reality in Scoliosis Correction Surgery: Efficiency and Accuracy in Pedicle Screw Instrumentation

**DOI:** 10.3390/medicina61040576

**Published:** 2025-03-24

**Authors:** Chia-Ning Chang, Chi-Ruei Li, Sian-Siang Liao, Chiung-Chyi Shen, Kai-Yuan Chen, Chung-Hsin Lee, Meng-Yin Yang

**Affiliations:** Department of Neurosurgery, Neurological Institute, Taichung Veterans General Hospital, No. 1650, Taiwan Boulevard, Sec. 4, Taichung City 40705, Taiwan; ning199001@gmail.com (C.-N.C.); fantastic1694@gmail.com (C.-R.L.); sonic5407@gmail.com (S.-S.L.); shengeorge@yahoo.com (C.-C.S.); bosminchen@gmail.com (K.-Y.C.); zacklee228@gmail.com (C.-H.L.)

**Keywords:** augmented reality, head-mounted display, hybrid operating room, surgical navigation, scoliosis surgery, spinal deformity

## Abstract

*Background and Objectives*: Recent advancements in spinal navigation methodologies, particularly augmented reality (AR) techniques, have significantly enhanced the precision of spinal instrumentation procedures. This study aimed to evaluate the efficacy of AR-assisted navigation in spinal instrumentation surgery for thoracolumbar scoliosis. *Materials and Methods*: This retrospective observational study included 10 patients with thoracolumbar scoliosis who met specific inclusion criteria and were recruited at a single medical center. Two neurosurgeons and one neuroradiologist used the Gertzbein–Robbins scale (GRS) for radiological evaluation. Preoperative and postoperative Cobb angles were measured to assess the correction of scoliosis. Overall, 257 screws were placed using the AR-assisted navigation system during thoracic and lumbar spinal deformity surgeries. *Results*: Among the 257 screws, 197 were placed in the thoracic spine and 60 in the lumbar spine, achieving an overall instrumentation accuracy of 98%. The preoperative Cobb angle of 69.5 ± 22.2° significantly improved to 10.1 ± 4.1° postoperatively. Regarding first-attempt screw placement accuracy, 97.4% of the screws in the thoracic spine (graded as GRS A or B) and 100% in the lumbar spine were placed with precision. Five grade C thoracic screws were identified, one of which required re-instrumentation. *Conclusions*: The AR navigation technique substantially improved the precision of spinal deformity surgery, with a high screw placement accuracy rate and significant scoliosis correction. The benefits of reduced attention diversion and an intuitive surgical experience suggest that AR technology could significantly improve spinal surgery practices and training programs, indicating potential for broader applicability in the future.

## 1. Introduction

Over the past decade, significant advancements in spinal navigation methodologies have revolutionized the precision of spinal instrumentation procedures, marking a substantial departure from traditional freehand techniques. Contemporary scholarly research has highlighted that navigation-assisted systems achieve accuracy rates ranging from 93.0% to 98.5%, a marked improvement over manual methods [1,2,3]. These systems have been particularly impactful in reducing the risk of neurological and vascular complications, especially in patients presenting with severe spinal deformities where anatomical complexities heighten procedural challenges [4]. Through offering detailed, real-time imaging and guidance, these technologies enhance the surgeon’s ability to achieve optimal pedicle screw placement and deformity correction. However, despite their advantages, navigation systems are not without limitations. A critical drawback lies in the potential to divert the surgeon’s focus from the operative field to external displays, requiring frequent shifts in attention and potentially increasing cognitive load. This limitation has prompted the development of augmented reality (AR) surgical innovations, designed to seamlessly integrate real-time anatomical feedback into the surgeon’s visual field. By overlaying critical imaging data directly onto the surgical site, AR systems eliminate the need to look away from the patient, thus maintaining focus while enhancing procedural accuracy. These advancements aim to bridge the gap between technology and workflow efficiency in spinal surgery.

The integration of advanced navigation techniques in spinal surgery has significantly reduced the amount of radiation to which surgical staff are exposed—a critical advantage highlighted in the literature [5,6]. Procedures utilizing navigation systems, particularly those involving 3D imaging, require less continuous radiation compared to conventional methods. Tajsic et al. [7] reported that the effective radiation dose from a 3D C-arm procedure is approximately one-fourth of that from traditional 2D fluoroscopy, underscoring the efficiency of modern imaging technologies. This substantial reduction is achieved by minimizing the need for repeated imaging cycles, as navigation systems provide an accurate, real-time visualization of anatomical structures. Similarly, a 2021 cohort study by Venier et al. [8] documented a notable decrease in cumulative radiation exposure when navigation-assisted techniques were employed, further validating the safety benefits of these systems. Through optimizing imaging protocols and reducing the reliance on fluoroscopy, navigation technologies not only enhance procedural accuracy but also protect surgical teams from the risks of prolonged radiation exposure. These advancements reflect a critical step forward in improving the safety of both patients and healthcare providers in the operating room, reinforcing the value of navigation systems in modern surgical practices.

This study examined thoracic and lumbar spinal deformities, presenting an AR-based technique for transpedicular screw instrumentation as a novel alternative to conventional intraoperative navigation systems in spinal surgery. By integrating AR technology within a cutting-edge hybrid operating room (hybrid OR), the system addresses the critical limitation of attention shifts that often occur with traditional navigation, where surgeons must frequently look away from the surgical field to external displays. The AR-based system overlays essential anatomical information and navigation guidance directly onto the surgeon’s visual field in real time, allowing for uninterrupted focus on the patient while ensuring precise screw placement and trajectory alignment. Coupled with the advanced imaging capabilities of the hybrid OR, this approach enhances real-time feedback and anatomical visualization, facilitating more accurate instrumentation and reducing potential errors. The seamless integration of augmented guidance and intraoperative imaging not only enhances surgical precision but also streamlines procedural workflows, providing a robust solution for managing complex spinal deformities. By leveraging AR technology, this innovative technique has the potential to redefine standards in spinal surgery, offering improved outcomes for both patients and surgeons.

## 2. Methods

### 2.1. Patient Collection

This study recruited patients diagnosed with thoracolumbar scoliosis who presented with complications characterized by one or more of the following criteria: (1) a curve angle exceeding 40°, (2) an annual increase in the curve angle of >10°, (3) the compression of visceral organs, and (4) limitations in socialization attributed to the deformity. In total, 10 patients were included. Demographic and surgical data for the 10 patients are presented in Table 1 and Table 2. All patients underwent pedicle screw implantation using an AR-assisted navigation system at a single medical center between 2022 and 2023. Patients who had previously undergone spine surgery and those with extreme BMI values, underlying neuromuscular disorders, or incomplete postoperative imaging data were excluded from the study. Given that the study primarily involved adolescent idiopathic scoliosis patients with a mean age of 22 years, osteoporosis was not considered a routine exclusion criterion. Additionally, all transpedicular screws were inserted by the same senior neurosurgeon to minimize operator-dependent variability. This study was approved on 9 June 2023 and was performed according to the Declaration of Helsinki. The requirement for informed consent was waived because of the retrospective nature of the study, and the analysis used anonymous clinical data.

### 2.2. Registration Procedures

The registration step was performed under general anesthesia with the patient in the prone position. The procedure involved midline exposure and spine dissection, followed by attaching a reference clamp to the spinous process for registration. Preoperative spinal imaging data were obtained in our hybrid operating room using an ARTIS icono robotic C-arm cone-beam computed tomography scanner (Siemens Healthcare GmbH, Forchheim, Germany). These data were integrated using commercial surgical planning software (OOOPDS, Taiwan Main Orthopaedic Biotechnology Co., Ltd., Taichung, Taiwan) and subsequently transmitted to a head-mounted device (HMD). This system presents axial, coronal, sagittal, and 3-dimensional reconstructed images, along with real-time navigation information. The surgeon can view anatomical projections in the surgical field through the HMD using transparent glasses.

### 2.3. Surgical Techniques

**Figure 1:**
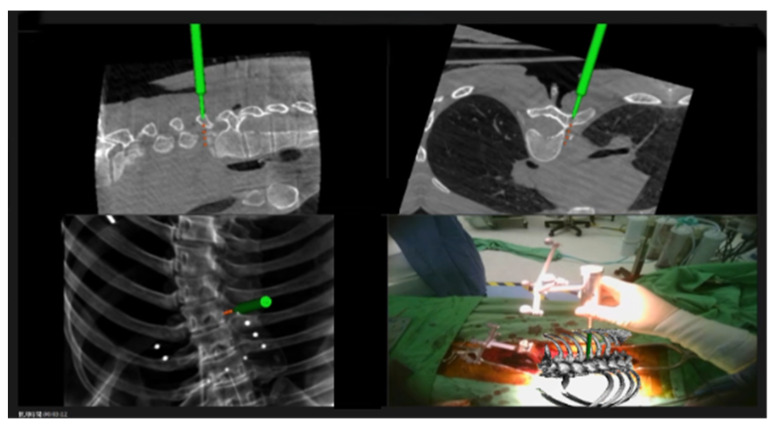
Real-time navigation information and a 3D augmented reality projection are displayed on the monitor and head-mounted glasses simultaneously. Surgeons can choose to display multiple views or only 3D anatomical images.

**Figure 2:**
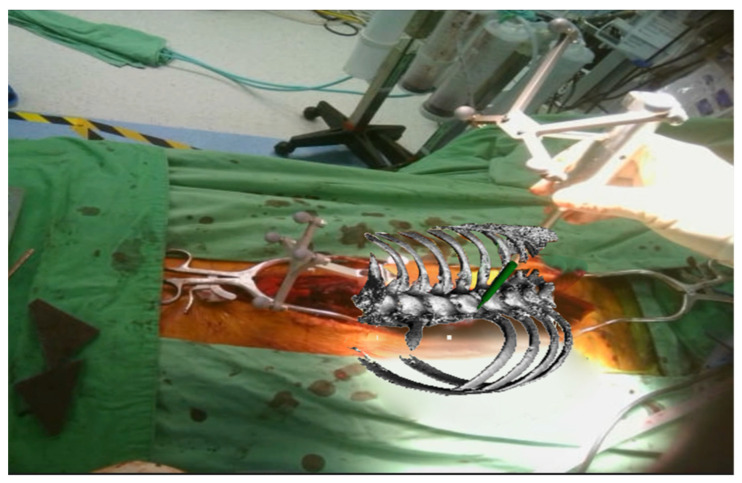
A 3D reconstructed image displayed through head-mounted glasses, including instrumentation tools in the projection.

Optimal screw placement was facilitated by the integration of 3D real-time navigation imaging with detailed anatomical projections directly visualized within the surgical field (Figure 1 and Figure 2). This advanced imaging technology enabled precise localization of the entry points and trajectory planning for pedicle screw insertion. The process began with the creation of an entry point using a high-speed burr, ensuring accuracy and reducing the risk of cortical breaches. Following this, a high-speed drill was employed to create a tunnel through the pedicle, carefully guided by the navigation system.

### 2.4. Radiological Evaluation

The screw placement accuracy was independently evaluated using the Gertzbein–Robbins scale (GRS) based on high-resolution computed tomography (CT) images [9]. In the first round, two experienced neurosurgeons (who were not the operating surgeon) independently assessed the accuracy of screw placement. If there were discrepancies between their evaluations, a neuroradiologist conducted a second-round review to make the final decision. The neuroradiologist also cross-checked the first-round results to ensure consistency. All evaluators were blinded to the surgical details to minimize bias and enhance the reliability of the assessments. The evaluation process involved categorizing any breaches in screw placement according to their location as medial, lateral, inferior, or superior. Grades A and B on the GRS were classified as indicating correctly positioned screws, reflecting their proximity to the pedicle walls without significant cortical breach. Additionally, Cobb angles were measured both preoperatively and postoperatively to quantify the degree of spinal deformity correction achieved, providing an objective assessment of surgical efficacy in scoliosis treatment.

## 3. Results

A total of 257 pedicle screws were placed in 10 patients, distributed as 197 screws in the thoracic spine and 60 in the lumbar–sacral spine. The screws were meticulously placed to address varying degrees of spinal deformities, with the thoracic spine requiring a higher density of instrumentation due to the complexity and rigidity of curvatures in this region. Preoperatively, the most severe spinal deformity observed was a Cobb angle of 69.5 ± 22.2°, reflecting the significant curvature associated with these conditions. Postoperatively, the Cobb angle was reduced to 10.1 ± 4.1°, indicating a substantial degree of correction achieved through the surgical intervention.

The accuracy of first-attempt screw placement in this study was notably high, with 97.4% of screws placed in the thoracic spine and 100.0% of screws in the lumbar spine being graded as GRS A or B (Table 3). This underscores the effectiveness of the surgical technique and navigation system employed. Among the 257 screws placed, 5 screws in the thoracic spine (1.9%) were graded as GRS C. Of these, one screw, accounting for 0.3% of the total screws, was identified as having a medial breach. This breach was detected intraoperatively through fluoroscopy and necessitated re-instrumentation to ensure proper placement and avoid potential complications. The remaining four GRS C screws (1.5%) presented with lateral breaches but did not require re-instrumentation.

None of the 10 patients in the cohort developed new postoperative neurological deficits, reflecting the precision of screw placement and the surgeons’ ability to address any deviations intraoperatively. Follow-up evaluations further validated the efficacy and safety of the procedure. During the follow-up period (ranging from 14 to 24 months), no cases of screw loosening, hardware failure, or infection were observed.

## 4. Discussion

This study aimed to evaluate the efficacy of an AR navigation technique in spinal surgery. In practice, this technique demonstrated remarkable accuracy for screw instrumentation: 97.4% for the thoracic spine and 100.0% for the lumbar spine, resulting in an overall accuracy of 98.0%. Furthermore, the significant correction observed in the postoperative Cobb angle, from 69.5 ± 22.2° to 10.1 ± 4.1°, highlights the technique’s effectiveness. These results affirm the high precision of AR navigation in spinal surgery and suggest its potential to improve surgical outcomes and patient safety.

It is generally acknowledged that medial screw misplacement leads to severe neurological deficits. In addition to iatrogenic neurological harm, screw malposition remains a predominant cause of subsequent revision surgeries. A review of the existing literature revealed that spinal surgery aided by navigational techniques, whether fluoroscopy or intraoperative 3D imaging, markedly enhances the precision of pedicular screw placement [10,11,12].

In contrast to ARSN systems, conventional optical navigation systems face technological constraints, primarily due to line-of-sight issues. These obstructions can obscure the tracking markers on surgical instruments, making them “invisible” to external cameras and disrupting the navigation process [13]. To overcome this challenge, HMD AR glasses can facilitate direct recognition of the navigation tool’s tracking marker, streamlining surgical operations. Additionally, the integration of HMD AR glasses eliminates the need for external reference monitors by enabling the transmission of all navigation data to the AR glasses and seamlessly merging these data with the anatomical view within the surgical field. In 2021, Molina et al. employed a technique similar to an AR navigation system involving an HMD approved by the Food and Drug Administration [14]. This approach shows promise as it helps surgeons maintain focus, thereby reducing attention shifts. Yahanda et al. [15] also reported nine cases involving the placement of 63 percutaneous thoracolumbar pedicle screws assisted by an HMD-equipped ARSN system that demonstrated promising accuracy, with a 100% rate of Gertzbein–Robbins grade A or B screw placement.

In spinal surgery, the precision of pedicle screw instrumentation is paramount. Several cadaveric and model studies have demonstrated that augmented reality surgical navigation (ARSN) achieves comparable or superior accuracy compared to intraoperative C-arm fluoroscopic guidance or freehand techniques [16,17,18,19,20,21,22,23,24]. Cao et al. [25] reported a higher screw accuracy and shorter instrumentation time in the ARSN group in a comparative cadaveric study. Similarly, a clinical study by Edström et al. [26], which involved 44 patients with thoracolumbar spinal deformities, found no significant difference in the operation time or correction rate between AR navigation and conventional methods. Previous studies have reported varying accuracy rates for freehand and robotic-assisted pedicle screw placement. According to a systematic review by Peng et al. (2020), freehand screw placement demonstrates an accuracy rate ranging from 85% to 95%, whereas robotic-assisted techniques achieve 90% to 98% accuracy in spine surgery [27]. Given the clarity of images obtained from radiographs, CT scans, and magnetic resonance imaging, AR technology has emerged as a promising candidate for deformity surgery, offering enhanced visualization and precision [28]. Our findings further support this, as we observed a first-attempt screw placement accuracy of 97.4% using the AR technique, which is consistent with the highest accuracy rates reported for robotic-assisted surgery. These results reinforce AR-assisted navigation as a viable and effective alternative to both freehand and robotic-assisted techniques in pedicle screw placement.

At present, mainstream AR applications in spinal surgery primarily use separate views, including axial, sagittal, and coronal images displayed on an HMD [14,16,28], rather than an intuitive 3D reconstruction projected directly onto the surgical field. While this AR technique reduces attention shifts due to head or visual movement, surgeons still need to reconstruct these images mentally, particularly the axial and sagittal views, to ensure an optimal screw trajectory. Conversely, our study reveals that the direct projection of these images onto the surgical field is more intuitive for surgeons. This method significantly increases spatial awareness and structural clarity, improving the surgical process. Therefore, it has emerged as the prime choice for deformity correction.

In our report on the efficiency of a novel technique, which is significantly influenced by the operator’s learning curve, we found that the mean screw placement times for the two most recent cases were 1 min and 26 s, with a range from 48 s to 3 min and 49 s. As proficiency with the AR-guided approach improves, we have observed promising enhancements in accuracy and efficiency for scoliosis surgeries. This average time compares favorably with the efficiency of previously reported pedicle screw placements, although existing data are somewhat limited, and the methods for timing these procedures vary. Bäcker et al. demonstrated a mean screw placement time of 8.6 ± 3.3 min for robot-assisted percutaneous screw placement, but only 4 out of their 46 cases involved scoliosis correction surgery [29]. Additionally, Butler et al. reported an average screw placement time of 3 min and 54 s using AR-assisted percutaneous pedicle screw placement in a minimally invasive approach [30]. While the studies mentioned above did not focus solely on scoliosis surgery, our results indicate a significant improvement in time efficiency despite the added complexity of deformed spinal alignment.

In contemporary practice, AR technology is predominantly employed in degenerative thoracolumbar instrumentation surgery. The existing literature, including case series, technical notes, and reviews, has primarily focused on the domain of thoracolumbar surgery [31,32,33,34,35,36,37,38,39,40]. Limited studies have addressed the application of AR techniques in cervical spinal surgery [39,40,41,42,43], scoliosis correction [26,28,44], and spinal tumor operations [45,46,47,48]. The clarity of preoperative or intraoperative imagery allows surgeons to identify critical vascular structures more accurately and reduce the risk of complications. A recent study by Lin et al. [48], which examined C1-C2 posterior instrumentation in patients with rheumatoid arthritis, revealed that microscope-based AR navigation provides a clear, real-time visualization of the vertebral artery and screw trajectory. With the continuous advancement of this innovative technique and the increasing familiarity of surgeons with the ARNS system, its application across diverse spinal surgeries is expected to expand.

The AR technique offers significant advancements in preoperative consultations by providing patients and their families with clearer and more realistic information regarding upcoming surgical procedures. In outpatient departments, an AR system integrated with a spine model can be used by neurosurgeons to provide a comprehensive explanation of surgical details. Furthermore, this technique has great potential for spinal surgery training programs, where it can serve as a valuable educational tool.

However, this novel surgical technique has some limitations. First, the sample size in this study was relatively small, with only 10 patients undergoing the procedure using head-mounted AR technology. This limited sample size affects the generalizability of the treatment outcomes. Additionally, no power analysis was conducted to determine whether the sample size was sufficient to detect significant differences. While this study serves as an initial exploration of the feasibility and potential benefits of AR-assisted screw insertion in scoliosis surgery, further research with a larger sample size and an appropriate power analysis is necessary to validate the accuracy and effectiveness of this technique. Second, the relatively short follow-up period (ranging from 14 to 24 months) may not be sufficient to assess long-term efficacy, even though no cases of loss of correction were observed during this period. Third, the surgeon must adapt to the display projected onto their retina, as factors such as interocular distance, focal length, binocular parallax, and equipment dislocation may interfere with the surgical procedure. Lastly, the high setup and maintenance costs remain a significant barrier to the widespread implementation of navigation systems.

## 5. Conclusions

This study demonstrated the potential of AR techniques for enhancing the precision of spinal deformity surgery. A total of 257 screws were accurately placed in the thoracic and lumbar spines, achieving an overall instrumentation accuracy rate of 98%. The AR technique offers potential benefits, including reduced attention shifts and a more intuitive surgical experience, making it a promising tool for future spinal surgeries and training programs. While our findings suggest that AR technology may contribute to advancements in surgical practices and education, further studies with larger sample sizes and comparative analyses are needed to validate its effectiveness and broader applicability.

## Figures and Tables

**Table 1:** Patients’ demographic data.

Demographic Factor	
Average age (years)	22.0
Average body mass index (kg/m^2^)	20.17
Sex (Male/Female)	1 (10%)/9 (90%)

**Table 2:** Data on instrumentation surgeries.

Case No.	Instrumented Segments	Largest Preoperative Cobb Angle (°)	Largest Postoperative Cobb Angle (°)
1	Left T2 and bilateral T3 to L2	72	9
2	Bilateral T2 to L4	98	6
3	Bilateral T2 to L2	45	5
4	Bilateral T5 to L5	60	9
5	Bilateral T2 to L2	85	13
6	Bilateral T3 to L2	78	14
7	Bilateral T2 to L3	72	6
8	Bilateral T3 to L3	52	6
9	Bilateral T1 to L3	75	15
10	Bilateral T2 to L5	124	16

**Table 3:** GRS results for screws placed in the L-spine and T-spine.

	Total	GRS A	GRS B	GRS C	Accuracy
T-spine	197	132	60	5	97.4%
L-spine	60	41	19	0	100.0%

GRS, Gertzbein–Robbins scale; T-spine, thoracic spine; L-spine, lumbar spine.

## Data Availability

Restrictions apply regarding the availability of these data. The data are not publicly available, but they are available from the corresponding author upon reasonable request and with permission from the Taichung Veterans General Hospital.

## References

[1] Qian B.P., Zhang Y.P., Qiao M., Qiu Y., Mao S.H. (2018). Accuracy of freehand pedicle screw placement in surgical correction of thoracolumbar kyphosis secondary to ankylosing spondylitis: A computed tomography investigation of 2314 consecutive screws. World Neurosurg..

[2] Abul-Kasim K., Ohlin A. (2011). The rate of screw misplacement in segmental pedicle screw fixation in adolescent idiopathic scoliosis. Acta Orthop..

[3] Hersh A., Mahapatra S., Weber-Levine C., Awosika T., Theodore J.N., Zakaria H.M., Liu A., Witham T.F., Theodore N. (2021). Augmented reality in spine surgery: A narrative review. HSS J..

[4] Jin M., Liu Z., Qiu Y., Yan H., Han X., Zhu Z. (2017). Incidence and risk factors for the misplacement of pedicle screws in scoliosis surgery assisted by O-arm navigation-analysis of a large series of one thousand, one hundred and forty five screws. Int. Orthop..

[5] Kim C.W., Lee Y.P., Taylor W., Oygar A., Kim W.K. (2008). Use of navigation-assisted fluoroscopy to decrease radiation exposure during minimally invasive spine surgery. Spine J..

[6] Ruatti S., Dubois C., Chipon E., Kerschbaumer G., Milaire M., Moreau-Gaudry A., Tonetti J., Merloz P. (2016). Interest of intra-operative 3D imaging in spine surgery: A prospective randomized study. Eur. Spine J..

[7] Tajsic T., Patel K., Farmer R., Mannion R.J., Trivedi R.A. (2018). Spinal navigation for minimally invasive thoracic and lumbosacral spine fixation: Implications for radiation exposure, operative time, and accuracy of pedicle screw placement. Eur. Spine J..

[8] Venier A., Croci D., Robert T., Distefano D., Presilla S., Scarone P. (2021). Use of intraoperative computed tomography improves outcome of minimally invasive transforaminal lumbar interbody fusion: A single-center retrospective cohort study. World Neurosurg..

[9] Gertzbein S.D., Robbins S.E. (1990). Accuracy of pedicular screw placement in vivo. Spine.

[10] Safaee M., Oh T., Pekmezci M., Clark A.J. (2019). Cone beam intraoperative computed tomography-based image guidance for minimally invasive transforaminal interbody fusion. J. Vis. Exp..

[11] Lian X., Navarro-Ramirez R., Berlin C., Jada A., Moriguchi Y., Zhang Q., Härtl R. (2016). Total 3D Airo^®^ navigation for minimally invasive transforaminal lumbar interbody fusion. BioMed Res. Int..

[12] Silbermann J., Riese F., Allam Y., Reichert T., Koeppert H., Gutberlet M. (2011). Computer tomography assessment of pedicle screw placement in lumbar and sacral spine: Comparison between freehand and O-arm based navigation techniques. Eur. Spine J..

[13] Mehbodniya A.H., Moghavvemi M., Narayanan V., Waran V. (2019). Frequency and causes of line of sight issues during neurosurgical procedures using optical image-guided systems. World Neurosurg..

[14] Molina C.A., Sciubba D.M., Greenberg J.K., Khan M., Witham T. (2021). Clinical accuracy, technical precision, and workflow of the first in human use of an augmented-reality head-mounted display stereotactic navigation system for spine surgery. Oper. Neurosurg..

[15] Yahanda A.T., Moore E., Ray W.Z., Pennicooke B., Jennings J.W., Molina C.A. (2021). First in-human report of the clinical accuracy of thoracolumbar percutaneous pedicle screw placement using augmented reality guidance. Neurosurg. Focus.

[16] Elmi-Terander A., Nachabe R., Skulason H., Pedersen K., Söderman M., Racadio J., Babic D., Gerdhem P., Edström E. (2018). Feasibility and accuracy of thoracolumbar minimally invasive pedicle screw placement with augmented reality navigation technology. Spine.

[17] Liebmann F., Roner S., von Atzigen M., Scaramuzza D., Sutter R., Snedeker J., Farshad M., Fürnstahl P. (2019). Pedicle screw navigation using surface digitization on the Microsoft HoloLens. Int. J. Comput. Assist. Radiol. Surg..

[18] Liu H., Wu J., Tang Y., Li H., Wang W., Li C., Zhou Y. (2020). Percutaneous placement of lumbar pedicle screws via intraoperative CT image-based augmented reality-guided technology. J. Neurosurg. Spine.

[19] Müller F., Roner S., Liebmann F., Spirig J.M., Fürnstahl P., Farshad M. (2020). Augmented reality navigation for spinal pedicle screw instrumentation using intraoperative 3D imaging. Spine J..

[20] Dennler C., Jaberg L., Spirig J., Agten C., Götschi T., Fürnstahl P., Farshad M. (2020). Augmented reality-based navigation increases precision of pedicle screw insertion. J. Orthop. Surg. Res..

[21] Yanni D.S., Ozgur B.M., Louis R.G., Shekhtman Y., Iyer R.R., Boddapati V., Iyer A., Patel P.D., Jani R., Cummock M. (2021). Real-time navigation guidance with intraoperative CT imaging for pedicle screw placement using an augmented reality head-mounted display: A proof-of-concept study. Neurosurg. Focus.

[22] Spirig J.M., Roner S., Liebmann F., Fürnstahl P., Farshad M. (2021). Augmented reality-navigated pedicle screw placement: A cadaveric pilot study. Eur. Spine J..

[23] Frisk H., Lindqvist E., Persson O., Weinzierl J., Bruetzel L.K., Cewe P., Burström G., Edström E., Elmi-Terander A. (2022). Feasibility and accuracy of thoracolumbar pedicle screw placement using an augmented reality head-mounted device. Sensors.

[24] Felix B., Kalatar S.B., Moatz B., Hofstetter C., Karsy M., Parr R., Gibby W. (2022). Augmented reality spine surgery navigation: Increasing pedicle screw insertion accuracy for both open and minimally invasive spine surgeries. Spine.

[25] Cao B., Yuan B., Xu G., Zhao Y., Sun Y., Wang Z., Zhou S., Xu Z., Wang Y., Chen X. (2023). A pilot human cadaveric study on accuracy of the augmented reality surgical navigation system for thoracolumbar pedicle screw insertion using a new intraoperative rapid registration method. J. Digit. Imaging.

[26] Edström E., Burström G., Persson O., Charalampidis A., Nachabe R., Gerdhem P., Elmi-Terander A. (2020). Does augmented reality navigation increase pedicle screw density compared to freehand technique in deformity surgery? single surgeon case series of 44 patients. Spine.

[27] Peng Y.N., Tsai L.C., Hsu H.C., Kao C.H. (2020). Accuracy of robot-assisted versus conventional freehand pedicle screw placement in spine surgery: A systematic review and meta-analysis of randomized controlled trials. Ann. Transl. Med..

[28] Sakai D., Schol J., Kawachi A., Sako K., Hiyama A., Katoh H., Sato M., Watanabe M. (2023). Adolescent idiopathic scoliotic deformity correction surgery assisted by smart glasses can enhance correction outcomes and accuracy and also improve surgeon fatigue. World Neurosurg..

[29] Bäcker H.C., Freibott C.E., Perka C., Weidenbaum M. (2020). Surgeons’ learning curve of renaissance robotic surgical system. Int. J. Spine Surg..

[30] Butler A.J., Colman M.W., Lynch J., Phillips F.M. (2023). Augmented reality in minimally invasive spine surgery: Early efficiency and complications of percutaneous pedicle screw instrumentation. Spine J..

[31] Burström G., Nachabe R., Homan R., Hoppenbrouwers J., Holthuizen R., Persson O., Edström E., Elmi-Terander A. (2020). Frameless patient tracking with adhesive optical skin markers for augmented reality surgical navigation in spine surgery. Spine.

[32] Fatima N., Massaad E., Hadzipasic M., Shankar G.M., Shin J.H. (2021). Safety and accuracy of robot-assisted placement of pedicle screws compared to conventional freehand technique: A systematic review and meta-analysis. Spine J..

[33] Burström G., Persson O., Edström E., Elmi-Terander A. (2021). Augmented reality navigation in spine surgery: A systematic review. Acta Neurochir..

[34] Farshad M., Fürnstahl P., Spirig J.M. (2021). First in man in-situ augmented reality pedicle screw navigation. N. Am. Spine Soc. J..

[35] Charles Y.P., Cazzato R.L., Nachabe R., Chatterjea A., Steib J.P., Gangi A. (2021). Minimally invasive transforaminal lumbar interbody fusion using augmented reality surgical navigation for percutaneous pedicle screw placement. Clin. Spine Surg..

[36] Bhatt F.R., Orosz L.D., Tewari A., Boyd D., Roy R., Good C.R., Schuler T.C., Haines C.M., Jazini E. (2023). Augmented reality-assisted spine surgery: An early experience demonstrating safety and accuracy with 218 screws. Glob. Spine J..

[37] Harel R., Anekstein Y., Raichel M., Molina C.A., Ruiz-Cardozo M.A., Orrú E., Khan M., Mirovsky Y., Smorgick Y. (2022). The XVS system during open spinal fixation procedures in patients requiring pedicle screw placement in the lumbosacral spine. World Neurosurg..

[38] Sommer F., Hussain I., Kirnaz S., Goldberg J.L., Navarro-Ramirez R., McGrath L.B., Schmidt F.A., Medary B., Gadjradj P.S., Härtl R. (2022). Augmented reality to improve surgical workflow in minimally invasive transforaminal lumbar interbody fusion—A feasibility study with case series. Neurospine.

[39] Rush A.J., Shepard N., Nolte M., Siemionow K., Phillips F. (2022). Augmented reality in spine surgery: Current state of the art. Int. J. Spine Surg..

[40] Li C.R., Shen C.C., Yang M.Y., Lee C.H. (2023). Intraoperative augmented reality in minimally invasive spine surgery: A case report. Asian J. Surg..

[41] Carl B., Bopp M., Saß B., Nimsky C. (2019). Microscope-based augmented reality in degenerative spine surgery: Initial experience. World Neurosurg..

[42] Carl B., Bopp M., Saß B., Pojskic M., Voellger B., Nimsky C. (2020). Spine surgery supported by augmented reality. Glob. Spine J..

[43] Onuma H., Sakai K., Arai Y., Torigoe I., Tomori M., Sakaki K., Hirai T., Egawa S., Kobayashi Y., Okawa A. (2023). Augmented reality support for anterior decompression and fusion using floating method for cervical ossification of the posterior longitudinal ligament. J. Clin. Med..

[44] McClendon J., Almekkawi A.K., Abi-Aad K.R., Maiti T. (2020). Use of Pheno room, augmented reality, and 3-rod technique for 3-dimensional correction of adolescent idiopathic scoliosis. World Neurosurg..

[45] Carl B., Bopp M., Saß B., Voellger B., Nimsky C. (2019). Implementation of augmented reality support in spine surgery. Eur. Spine J..

[46] Sommer F., Hussain I., Kirnaz S., Goldberg J., McGrath L., Navarro-Ramirez R., Waterkeyn F., Schmidt F., Gadjradj P.S., Härtl R. (2022). Safety and feasibility of augmented reality assistance in minimally invasive and open resection of benign intradural extramedullary tumors. Neurospine.

[47] Tigchelaar S.S., Medress Z.A., Quon J., Dang P., Barbery D., Bobrow A., Kin C., Louis R., Desai A. (2022). Augmented reality neuronavigation for en bloc resection of spinal column lesions. World Neurosurg..

[48] Lin M.S., Huang C.W., Tsou H.K., Tzeng C.Y., Kao T.H., Lin R.H., Chen T.Y., Li C.R., Lee C.Y. (2023). Advances in surgical treatment for atlantoaxial instability focusing on rheumatoid arthritis: Analysis of a series of 67 patients. Int. J. Rheum. Dis..

